# LDA-based topic modeling for COVID-19-related sports research trends

**DOI:** 10.3389/fpsyg.2022.1033872

**Published:** 2022-11-14

**Authors:** Jea Woog Lee, YoungBin Kim, Doug Hyun Han

**Affiliations:** ^1^Intelligent Information Processing Lab, Chung-Ang University, Seoul, South Korea; ^2^Department of Image Science and Arts, Chung-Ang University, Seoul, South Korea; ^3^Department of Psychiatry, Chung-Ang University School of Medicine, Seoul, South Korea

**Keywords:** sport, COVID-19, research trend, topic modeling, data science, LDA algorithm

## Abstract

**Introduction:**

The COVID-19 pandemic could generate a turning point for introducing a new system for sports participation and business. The purpose of this study is to explore trends and topic structures of COVID-19-related sports research by analyzing the relevant literature.

**Methods:**

Sports studies related to COVID-19 were collected in searching international academic databases. After the pre-processing step using the refinement and morpheme analysis function of the Net Miner program, topic modeling and social network analysis were used to analyze Journal Citation Reports found using the search term ‘COVID-19 sports’.

**Results:**

As a result, this study used subject modeling to reveal important potential topics in COVID-19-related sports research articles. ‘Sports participation’, ‘elite players’, and ‘sports industry’ were macroscopically classified, and detailed research topics could be identified from each division.

**Conclusion:**

This study revealed important latent topics from COVID-19-related sports research articles using topic modeling. The results of the research elucidate the structure of academic knowledge on this topic and provide guidance for future research.

## Introduction

On 11 March 2020, the World Health Organization (WHO) declared the highest epidemic alert for COVID-19 as a ‘Pandemic’ (Taghizadeh-Hesary and Akbari, 2020). The WHO has argued that the global spread of COVID-19 is not just a public health crisis but rather a crisis that affects politics, economics, society, and culture (Azorín, 2020). Countries around the world are seeking an active response to overcome the pandemic’s negative impacts. The COVID-19 pandemic could generate a turning point for introducing a new system. As of July 2022, several countries are in a state of lull (people have returned to normal life, lockdowns have ended, etc.) but have not completely recovered (Contractor, 2022).

Sports were similarly affected by the pandemic. The response of national sports leagues varied, but international leagues experienced a complete shutdown of sporting events at all levels (Bas et al., 2020). Throughout the pandemic, professional sports leagues both restricted and completely prohibited spectators, and large events such as the Olympics and various sports leagues were canceled or postponed (Elkhouly et al., 2022). According to Füzéki et al. (2020), the most serious sports-related problem caused by the pandemic is that people have almost no opportunities for exercise and physical activity in their daily life. The impact of COVID-19 on sports continues to be confirmed by evidence from various studies.

Nevertheless, athletes and the general population have proposed solutions for participating in sports during the pandemic, but they face several difficulties (Romero-Blanco et al., 2020). Evidence of the impact of these innovative initiatives has only recently begun to emerge (Ebersberger and Kuckertz, 2021). The current situation in the sports world, which has changed rapidly after the pandemic, will likely endure (Doherty et al., 2022). It is necessary to predict and prepare for the future of sports while considering this possibility. The publication and dissemination of academic articles about COVID-19 have become important for identifying the various impacts of COVID-19 (Älgå et al., 2020). Investigation and discussion of these impacts are being published in various scholarly works, such as original articles, reviews, proceedings, reports, etc. (Liu et al., 2021). According to the Web of Science, from January 2020 to July 2022, a number of COVID-19-related studies were published. Sonmez and Codal (2021) reported that this amount of scientific publication had never been observed in such a short time in any scientific field. Due to the need to overcome COVID-19 and examine the status and issues of this period, several studies have been conducted with a macro and exploratory approach for policy-making and strategic planning of COVID-19 research.

The exponential publishing of COVID-19-related studies in a short period includes studies that look at the impacts of COVID-19 on sports. Extensive research is being conducted on various aspects of the relationship between COVID-19 and sports(Älgå et al., 2020; Dastani and Danesh, 2021; Zhu and Lei, 2022). Scientists and researchers around the world have been conducting significant research on aspects of and methods for investigating, problem-solving, and planning related to COVID-19, the results of which have been published in peer-reviewed journals (Kemp et al., 2021; Teare and Taks, 2021). The rise in the number of global publications on COVID-19 has been driven by researchers’ efforts to fully understand the virus and the pandemic it caused (Samuel et al., 2020).

However, researchers may experience confusion while navigating the current literature (Taneja et al., 2021). A rapid assessment of a dynamic research focus, such as COVID-19, where the amount of evidence is increasing at an impressive rate, requires scoping and systematic review (Maulana, 2020). However, a comprehensive assessment of all available scientific publications on COVID-19 is lacking. We, therefore, aimed to use a biblio-based approach to explore the published scientific literature on COVID-19, evaluate relevant topics, and map the evolution of research through the stages of the COVID-19 pandemic.

Topic modeling has been increasingly applied to scholarly works. The application of topic modeling can discover hidden subject areas and identify the current and future direction of a research field (Jiang et al., 2016). Articles contain important sources of theories and information of each articles, such as abstracts and keywords, but an analysis process that can effectively extract them is needed (Abuhay et al., 2018).

Topic modeling is an important technique for searching for useful information in unstructured text data extracted from various documents such as scholarly articles, reports, and news articles (Ramage et al., 2009). The exploration and extraction of information from a large volume of articles is useful for identifying current research trends. In addition, these processes can stimulate new topic ideas for researchers and experts and contribute to guiding future research (Reisenbichler and Reutterer, 2019).

However, most previous COVID-19-related studies of sports have only analyzed either newspapers or social media platforms, such as Twitter and Facebook (González et al., 2021; Naraine and Bakhsh, 2022). Although many studies focus on problems in sports caused by the COVID-19 pandemic, few have used bibliographic analysis. This study used topic modeling and social network analysis tools to derive the topic domain and visualization map of ‘Sports-COVID-19’.

Topic modeling discovers specific interconnections of topics by identifying the similarity between research articles (Kumari et al., 2021). The latent Dirichlet allocation (LDA) topic modeling technique revealed the latent knowledge dimension and structures in ‘Sports-COVID-19’ articles. We also used degree centrality and 2-mode social network analysis to analyze visualization networks of important keywords from publications. Using these various techniques, this study discovered important knowledge domains in the ‘Sports-COVID-19’ research field.

## Materials and methods

### Research model

Research trends related to ‘COVID-19 and sports’ were analyzed using topic modeling and social network techniques. Various programs can be used for analysis, such as Net Miner, NodeXL, UNICET, and Net Draw. In particular, Net Miner can interactively perform topic modeling and social network analysis. It is possible to visualize the topic modeling results expressed in keyword text as a 2-mode network through social network analysis (Rashed et al., 2019). The Net Miner ver. 4.2 program (Cyram Inc., Seongnam) was used for the entire process of data processing (data pre-processing and data analysis for results and visualization). First, the papers to be analyzed were extracted, the relationship between major keywords was understood through frequency analysis and centrality analysis, and the main topics studied were derived through topic modeling.

### Data collection

**Figure 1:**
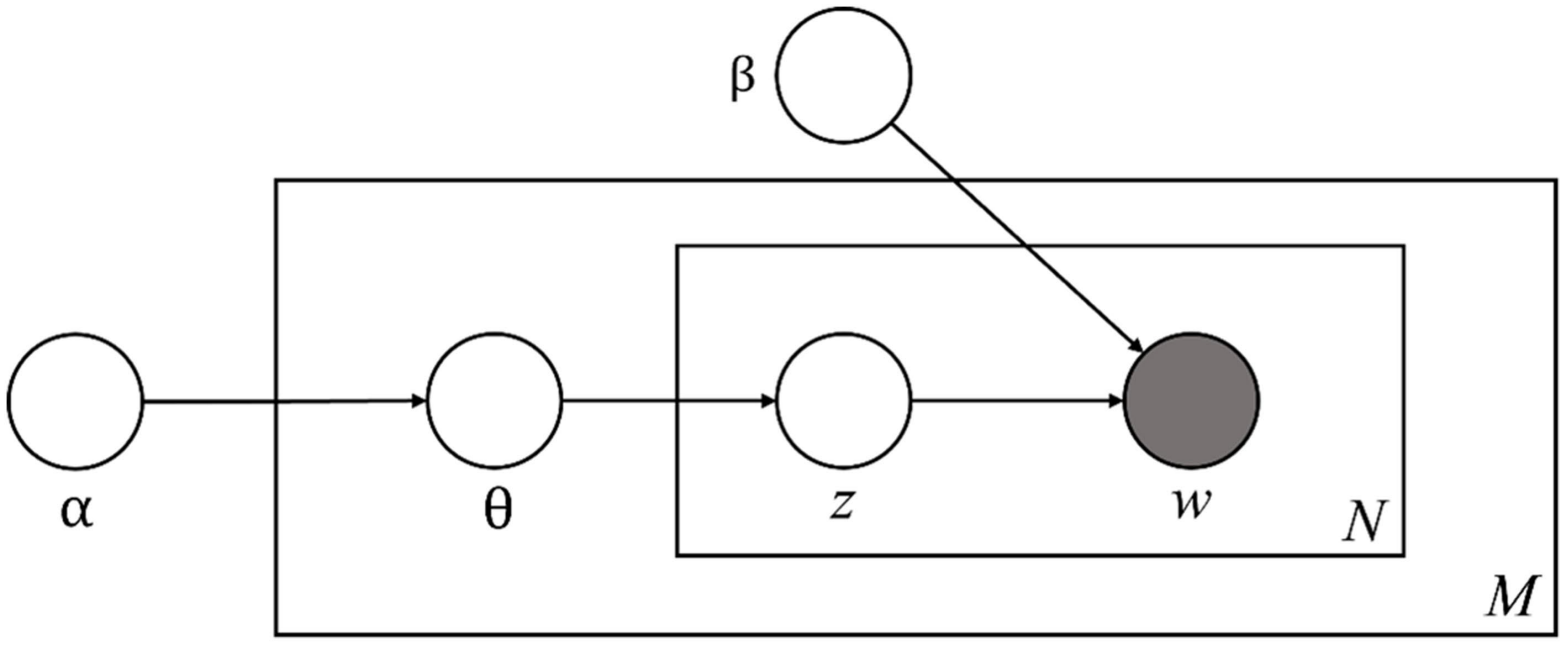
Probabilistic graphical model of latent Dirichlet allocation α: a parameter that represents the Dirichlet prior for the document topic distribution, β: a parameter that represents the Dirichlet for the word distribution, θ: a vector for topic distribution over a document d, *z*: a topic for a chosen word in a document, *w*: specific words in *N*, *M*: the length of documents, *N*: the number of words in the document.

Sports studies related to the COVID-19 pandemic were selected by searching international academic databases. Studies that were published internationally were included in this study. Articles were collected from the following indexes: Science Citation Index, Science Citation Index Expanded, Social Sciences Citation Index, and Arts & Humanities Citation Index. Keywords were used in the searches, including ‘Sport’, ‘Sports’, ‘Exercise’, ‘Physical Activity’, and ‘Leisure’, which were established by mixing ‘Sports’, and ‘COVID-19’ terms; further, ‘SARS-CoV-2’, ‘Corona’, and ‘Pandemic’ were added to limit the search to studies related to COVID-19 and sports. The ‘COVID-19 and sports’ research data used for this study included over 1,604 studies from 2020 to July 2022. Among the 1,604 papers searched in international databases, 157 duplicate papers were excluded. A total of 663 papers were excluded based on a review of their title, keywords, and abstract. A total of 267 papers not related to sports and 314 not related to COVID-19 were excluded. A total of 82 papers that were not journal articles (i.e., news articles, letters to editors, research reports, conference proceedings, and books) and whose abstracts were not available or were written in languages other than English were excluded. The research flow and procedure in this study are shown in Figure 1.

### Data pre-processing

The collected data are sentences in natural language, such as theories, knowledge, and opinions. Therefore, since each sentence itself cannot be used for analysis, it is necessary to perform a step of extracting each sentence as an individual word that can be analyzed (W. Zhao et al., 2015). In this study, the pre-processing step of extracting nouns explaining the core topic of the studies was carried out using the refinement and morpheme analysis function of the Net Miner program. A process was performed to remove words whose meanings created difficulties in explaining the meaning of the collected thesis among the extracted words. First, ‘stop words’ that do not have actual meanings, such as the conjunctions ‘because’, ‘but’, ‘however’, and ‘like’ were removed. In addition, words with an appearance frequency below six or that appeared in 20 or fewer articles were removed because they were judged inappropriate to explain the research flow according to the derivation of the results. On the other hand, the TF-IDF index is a numerical value indicating the representativeness of a single document based on the frequency of its appearance in a specific document (Kim and Gil, 2019). That is, in the case of a word with a low TF-IDF value, even though the overall frequency of appearance was high, if it was derived from the research results, it was judged that it could not be meaningfully interpreted and was deleted. In this study, the TF-IDF value deletion criterion was set to.05 or less in consideration of the total number of papers and the total number of extracted words (D. Kim et al., 2019). In addition, the process of unifying different marked words with the same meaning and replacing synonyms and synonyms with representative keywords was performed.

### Topic modeling

Topic modeling can be used to expose hidden topics in document sets. This analysis technique has been widely used has been widely used in academia to analyze text documents by applying text mining techniques (Ramage et al., 2009). One of the topic modeling algorithms, latent Dirichlet allocation (LDA), calculates a specific number of topics by considering the probability distribution of terms related to the topic (Rashed et al., 2019). Articles contain several topic-related terms that must be extracted and categorized from the appropriate text to explore the area of knowledge covered in the article. Topic modeling extracts topics by applying relevant keywords that help to recognize knowledge structures and patterns in research articles (Kumari et al., 2021). Figure 1 illustrates the graphical model of LDA (Debnath et al., 2020).

The probability calculation formula is as follows (Debnath and Bardhan, 2020).


$$p\left(D|\alpha ,\beta \right)=\overset{M}{\underset{d=1}{\prod }}\int p\left(\theta_{d}|\alpha \right)\left(\overset{N_{d}}{\underset{n=1}{\prod }}\underset{Z_{dn}}{\sum }p\left(Z_{dn}|\theta_{d}\right)p(W_{Zn}|Z_{dn},\beta )\right)d\theta_{d}$$

Figure 1 shows a probabilistic graphical model of LDA, where the boxes in Figure 1 are ‘plates’ representing replicates. The outer plate represents the document (M) and the inner plate represents the repeatedly selected topics (z) and words (w) within the document (N). ‘ϴ’ is the topic distribution of the document. That is, ‘α’ and ‘β’ are the two hyperparameters of the Dirichlet distribution. Since LDA cannot determine the number of topics on its own, the third hyperparameter is the ‘number of topics’ the algorithm will discover. To derive reasonable results, the alpha and beta values that determine the appropriate number of topics and keywords constituting each topic must be established (Song et al., 2019). Our judgment was needed to roughly estimate the total number of topics under each category through an iterative process of reading (W. X. Zhao et al., 2011). To evaluate how well each topic is clustered, we used a topic consistency metric. The coherence coefficient is an indicator that can evaluate how well the clusters are classified (O’callaghan et al., 2015). A value close to 1 means better clustering. Well-clustered means that it is far from other clusters and that data within the same cluster are well-clustered together. For a subject t characterized by a high-order word set Wt (a fixed number of high-order words or all words whose probability exceeds a predefined threshold), coherence is defined as (Mimno et al., 2011):


$$c\left(t,W_{t}\right)=\underset{w_{1}\neq w_{2},w_{1},w_{2}\in W_{t}}{\sum }\log\frac{d\left(w_{1},w_{2}\right)+\varepsilon }{d\left(w_{1}\right)}$$

Where d(wi) is the number of documents containing wi and d(wi, wj) is the number of documents in which wi and wj occur simultaneously, a smoothing factor is usually set to 1 or 0.01. Coherence and word co-occurrence statistics were commonly used for the initialization of LDA parameters (O’callaghan et al., 2015). By inputting alpha and beta values (0.01 to 0.99) of all ranges in the coherence formula, alpha (0.16) and beta (0.34) values corresponding to the optimal perplexity index were derived. Iteration was set to 5,000.

### Social network analysis

**Figure 2:**
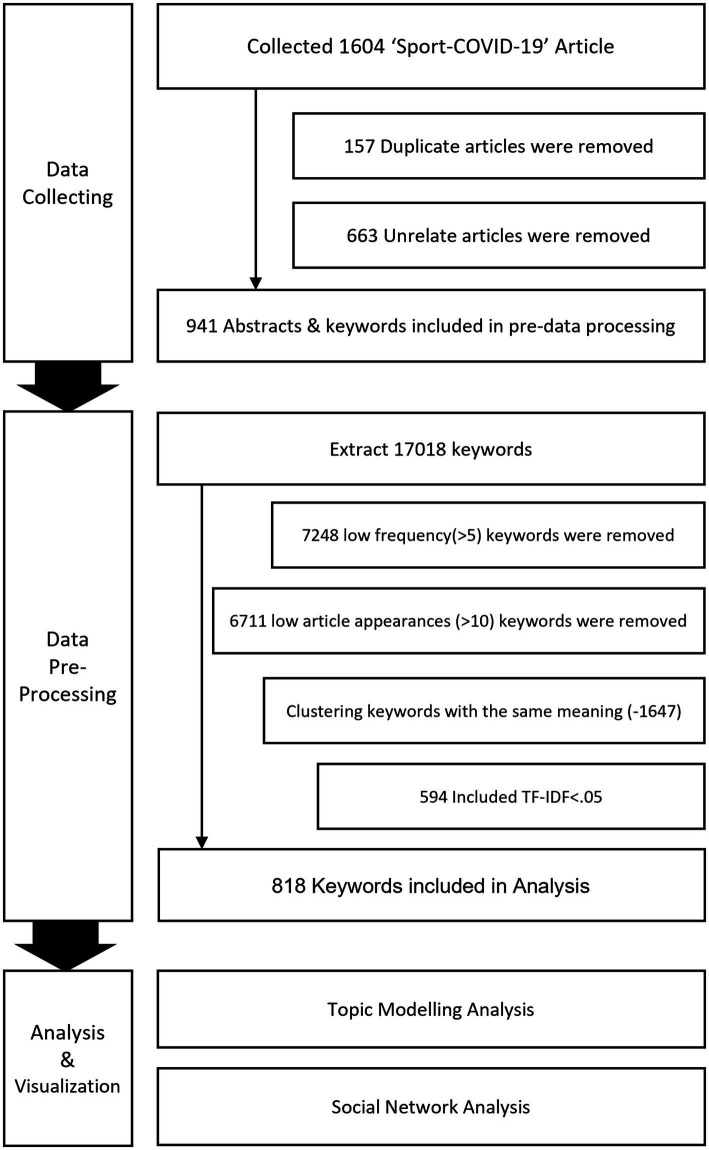
Research flow.

Social network analysis (SNA) is a method used to model the relationship between individuals or groups as nodes and links and to analyze the structure of the network or the process of diffusion and evolution (Chang, 2018). SNA is applied in various fields, such as society, humanities, information, engineering, chemistry, biology, and physics (Rueda et al., 2007). In this study, a 2-mode social network analysis technique was used to derive the topic modeling result for ‘COVID19-sport’ articles in the form of a visualization. The 2-mode network reveals which two other node types are connected in the map. It is used to check the connection structure between keywords belonging to each topic area and keywords that are duplicated in one or more topics (Borgatti, 2009). It has the advantage of being able to visualize and examine the group structure of the topic modeling results and the characteristics of keywords that penetrate the group. It was adopted as the analysis technique for this study. All procedures of this research method are shown in Figure 2.

## Results

### Results of topic modeling

Topic 1 is one of three subject areas related to ‘sports participation-lifestyle change relatedness’. The main keywords are as follows: ‘leisure’, ‘contents’, ‘satisfaction’, ‘service’, ‘environment’, ‘learning’, ‘coaching’, ‘course’, ‘application’, ‘technology’, ‘distance’, ‘experience’, ‘platform’, ‘training’, ‘virtual’, ‘reality’, ‘metaverse’, ‘acceptance’, and ‘intention’. This subject area is defined as research related to non-face-to-face sports participation in response to the COVID-19 pandemic. Specifically, it proposes virtual reality, metaverse platforms and base devices, contents, and online systems, and includes research to explore user reactions such as acceptance, intention, and satisfaction.

Topic 5 is the second topic among three subject areas related to ‘Sports participation-lifestyle change relatedness’. The main keywords are ‘confinement’, ‘behavior’, ‘adult’, ‘adolescent’, ‘increase’, ‘lifestyle’, ‘population’, ‘school’, ‘behavior’, ‘weight’, ‘habit’, ‘type’, ‘quarantine’, ‘decrease’, and ‘status’. This subject area is defined as lifestyle changes created by restrictions on participation in sports due to COVID-19. Specifically, it includes adult sports club activities and individual exercise, changes in hobbies due to the break in youth participation in school sports, weight gain, and decreased quality of life.

Topic 7 was discovered as the last subject area related to ‘Sports participation-physical and psychological symptoms’, and the main keywords are ‘distress’, ‘blue’, ‘capacity’, ‘behavior’, ‘sport club’, ‘stress’, ‘wellbeing,” ‘activity’, ‘depression’, ‘opportunity’, ‘performance’, ‘decrease’, ‘participants’, ‘relationship’, and ‘mood’. This subject area was discovered to deal with psychological problems caused by the restrictions placed on individuals and sports clubs due to COVID-19. Some factors that appeared in the topic. COVID blue, depression, stress, mood, etc., included the relationship between sports participation restriction and psychological factors.

Topic 2 is one of two subject areas related to ‘sports performance-inefficient performance improvement’, and the main keywords are ‘control’, ‘body’, ‘adult’, ‘capacity’, ‘function’, ‘outcome’, ‘performance’, ‘strength’, ‘rehabilitation’, ‘adolescent’, ‘coach’, ‘quality’, ‘muscle’, ‘training’, and ‘session’. This subject area is defined as research aimed at responding to a training environment that has become more difficult during the COVID-19 pandemic. Participants were athletes who ranged from teenagers to adults, and training included physical fitness, functional training, and rehabilitation training. It includes proposals of training systems, protocols, and studies demonstrating effectiveness for efficient training within a given period.

Topic 8 appeared as the second topic area related to ‘Sports performance-injury and rehabilitation’, and the main keywords are ‘mask’, ‘injury’, ‘player’, ‘incidence’, ‘case’, ‘performance’, ‘aerobics’, ‘medical’, ‘contact’, ‘coach’, ‘youth’, ‘support’, ‘fracture’, ‘rehabilitation’, and ‘team’. This topic area deals with sports medicine, with a focus on performance changes caused by various situations, such as lack of training due to COVID-19, wearing a mask, exercise function, aerobics, various injuries, and rehabilitation issues.

Topic 3 is one of two topic areas related to ‘Sport events-game stopped’. The main keywords are ‘game’, ‘Olympic’, ‘postpone’, ‘league’, ‘decrease’, ‘season’, ‘event’, ‘spectator’, ‘problem’, ‘crisis’, ‘interruption’, ‘without’, ‘stadium’, ‘outbreak’, and ‘reduce’. This subject area is defined as the discourse of crisis phenomena in mega-sports events, such as professional sports and the Olympics, due to COVID-19. Specifically, it includes a review of crisis situations, such as league suspension, reduction in spectators, prohibition of spectators, and reduction, postponement, and cancellation of competitions.

Topic 4 is one of two topic areas related to ‘Sport events-disconnection between player and fans’. The main keywords are ‘player’, ‘fan’, ‘communication’, ‘platform’, ‘smartphone’, ‘metaverse’, ‘virtual’, ‘reality’, ‘broadcasting’, ‘streaming’, ‘online’, ‘contents’, ‘YouTube’, ‘promotion’, and ‘strategy’. This subject area is focused on issues related to technical, social, and business aspects of non-face-to-face sports viewing. It includes online services for communication between players and fans, technology for non-face-to-face real-time broadcasting, such as the metaverse, virtual reality, and smartphones, and public relations strategies.

Topic 6 was discovered as the only subject area related to ‘Sport events-economic & business’, and the main keywords are ‘entrepreneurship’, ‘institutional’, ‘response’, ‘management’, ‘crisis’, ‘suggestion’, ‘ecosystem’, ‘strategy’, ‘financial’, ‘policy’, ‘industrial’, ‘plan’, ‘implicant’, ‘decrease’, ‘facility’, ‘employee’, and ‘clogging’. This subject area covers sports-related economic and business problems and responses due to COVID-19. It explains the contraction and crisis of the sports industry ecosystem, such as facility closures, deficits, and job loss. Topics also included policy proposals applicable to the pandemic environment, business strategy, and risk management as solutions to these problems (Table 1).

**Table 1:** Result of topic modeling.

Topic-1	Topic-2	Topic-3	Topic-4	Topic-5	Topic-6	Topic-7	Topic-8
Sports Participation-life style change relatedness’	Sports performance-inefficient performance improvement	Sport events-game stopped	Sport events-disconnection between player and fans	Sports Participation-life style change relatedness	Sport events-Economic & Business	Sports Participation-physical and psychological symptoms	Sports Performance-injury and rehabilitation
Leisure	Control	Game	Player	Confinement	Entrepreneurship	Distress	Mask
Contents	Body	Olympic	Fan	Behavior	Institutional	Blue	Injury
Satisfaction	Adult	Postpone	Communication	Adult	Response	Capacity	Player
Service	Capacity	League	Platform	Adolescent	Management	Behavior	Incidence
Environment	Function	Decrease	Smartphone	Increase	Crisis	Sport club	Case
Learning	Outcome	Season	Metaverse	Lifestyle	Suggestion	Stress	Performance
Coaching	Performance	Event	Virtual	Population	Ecosystem	Wellbeing	Aerobics
Course	Strength	Spectator	Reality	School	Strategy	Activity	Medical
Application	Rehabilitation	Problem	Broadcasting	Behavior	Financial	Depression	Contact
Technology	Adolescent	Crisis	Streaming	Weight	Policy	Opportunity	Coach
Distance	Coach	Interruption	Online	Habit	Industrial	Performance	Youth
Experience	Quality	Without	Contents	Type	Plan	Decrease	Support
Platform	Muscle	Stadium	Youtube	Quarantine	Implicant	Participants	Fracture
Training	Training	Outbreak	Promotion	Decrease	Decrease	Relationship	Rehabilitation
Virtual	Session	Reduce	Strategy	Status	Facility	Mood	Team
Reality					Employee		
Metaverse					Clogging		
Acceptance							
intention							

### Social network analysis for visualization

**Figure 3:**
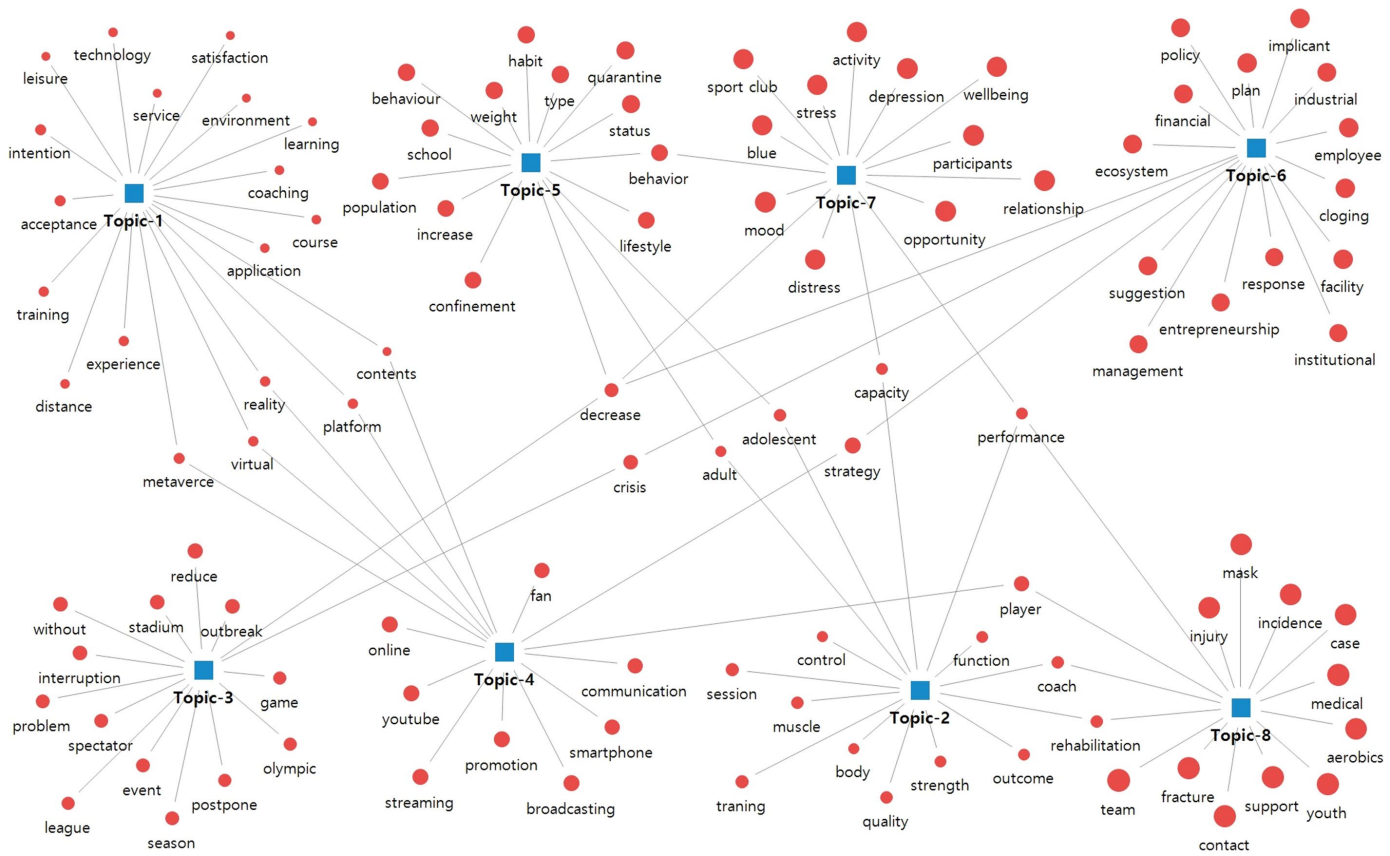
Semantic keywords network of topic modeling.

The mediating role of some keywords was confirmed through a network map that visualized the results of topic modeling. The keywords ‘metaverse’, ‘virtual’, ‘reality’, ‘platform’, and ‘contents’ are included in both Topic 1 (sports participation) and Topic 4 (pro sports & sports events). It is possible to check the vitalization of research on non-face-to-face spectating of sports and participation in sports due to COVID-19. The attempt to address sports-related social and economic issues caused by diseases through technology was verified with topics and keywords. On the other hand, the keyword ‘decrease’ encompasses situations in which physical activity, business profits, and sports opportunities are inevitably reduced. The response ‘strategy’ for various ‘crises’ caused by COVID-19 can be confirmed through a mediation network between topics. In addition, ‘adult’, ‘adolescent’, ‘capacity’, ‘player’, ‘rehabilitation’, ‘coach’, and ‘performance’ are important keywords that mediate issue topics related to amateur and elite sports participation and training. The results of visualizing the semantic network of 8 keywords in the topic–keyword map are shown in Figure 3.

## Discussion

We think the eight topics extracted from topic modeling could be classified into three areas: sports participation, sports performance, sports events including pro-leagues, Olympics, and sports business. The sports participation topic could include an exploration of alternatives, lifestyle change relatedness, and physical and psychological symptoms. The sports performance topic could include inefficient performance improvement and injury and rehabilitation. The sports events could include games stopped, disconnection between players and fans, and economics and business.

At first, sports participation might be reduced due to lifestyle changes in response to the COVID-19 pandemic (Buldú et al., 2020; DiFiori et al., 2021; Drewes et al., 2021). Reduced sports activity and participation could induce physical and psychological adverse symptoms, including distress, feeling blue, stress, and depressed mood (Qi et al., 2020; Giessing et al., 2021; Sa et al., 2021). For the compensation of withdrawn sport participation and overcoming adverse physical and psychological events, the exploration for alternative activities or healing methods could be tried in online contact technology with a variety of content on different platforms using virtual reality and metaverse technologies. Research issues related to ‘sports participation’ found in topics 1, 5, and 7 show the flow of publications focused on the problem of declining sports participation due to COVID-19 within a short period of 3 years (Pedersen, 2022). Society has experienced the rapid development of science and technology in the process of overcoming the COVID-19 pandemic (Williams, 2020). In the process of turning a crisis of participation into an opportunity in sports, a continuous approach to research on the convergence of devices, platforms, and virtual and online environments is predicted.

Second, improving sports performance in all sports may be significantly delayed during the COVID-19 pandemic. It has been difficult to effectively improve sports performance during the COVID-19 pandemic (Makarowski et al., 2020; Purwanto et al., 2021; Washif et al., 2021; Moore et al., 2022). In addition, this study demonstrated that inefficient athletic performance training caused by COVID-19 could worsen athletic injuries and prolong the rehabilitation period (Seshadri et al., 2021; Moore et al., 2022). Topic 2 and 8, ‘sports-performance’, could not discover potential rehabilitation methods and solutions for these problems. A few studies of training and rehabilitation issues in the COVID-19 environment were identified. This research predicted a limitation of experimental research that can only be carried out in direct contact. However, as the current atrophied research environment is resolved, alternative training, rehabilitation, and parallel methods with offline training may be developed.

Sports events, including pro-sports leagues and the Olympics, were canceled due to the COVID-19 pandemic. During that period, the number of sports games and events abruptly decreased, and the relationship between players and fans was broken (Chan et al., 2021; Cho et al., 2021; Kaplanidou et al., 2021; Kwon and Kwak, 2022). As a result, several sports businesses might be close to bankruptcy. To compensate for this crisis, sports organizations and businesses may invest in digital technologies that allow spectators to view games using virtual reality and metaverse (Ryall and Edgar, 2022; Smith and Skinner, 2022; Uslu, 2022). Topics 4 and 6 recognize that the pandemic has led to a new paradigm in sports and suggest a new direction to deal with the problems the pandemic has created. These proposals can potentially apply business and media strategies to the sports industry after the pandemic (Moritz et al., 2021). By examining the reactions of consumers and users in the sports industry, the effectiveness on economic feasibility can be proven.

Taken together, current topic modeling suggests that altered lifestyle, sports performance enhancement methods, and sports event business policy due to the COVID-19 pandemic would encourage the adaptation of digital facilities to sports areas. Future sports participation may be closely associated with online contact technology (Batez, 2021; Hurley, 2021). In addition, various digital platforms should be used for sports performance enhancement (Parker et al., 2021). The policy of sports business may actively adapt to both online technology and digital platforms (Ratten and Thompson, 2021; Skinner and Smith, 2021). In the future, the result of topic modeling can be further developed with relevant follow-up research. Analyzing large data sets from research articles has the potential to identify traits of hidden issues and training the topic modeling on data could generate valuable insight. The results of COVID-19-related sports research would be a useful resource for developing future sports policy and infrastructure. Importantly, we need the means to address all of the limitations and problems identified in research using topic modeling methods (Table 1).

## Conclusion

This study investigated the research trends and structure of COVID-19-related sports research by analyzing the relevant literature. Analyses of large volumes of knowledge can help researchers understand the productivity, classification, and growth of research fields. This knowledge can inform researchers of the structure of academic knowledge in a field and provide guidance for future research. This study revealed important latent topics from COVID-19-related sports research articles using topic modeling. This study has some limitations. Although the association between COVID-19 and the sports research field has been confirmed, further analysis could be performed in an independent research area. Topic modeling of ‘sports participation’, ‘sports performance’, and ‘sport events’ classified in this study could be performed individually. By subdividing the scope of the research, it would be possible to conduct a more systematic review of COVID-19-related sports research. However, despite these limitations, this study provides insight into the post-pandemic status of sports and the direction of relevant research.

## Data availability statement

The raw data supporting the conclusions of this article will be made available by the authors, without undue reservation.

## Author contributions

JL, YK, and DH contributed to data collection and pre-processing. JL and DH analyzed the data. All authors participated in writing of the manuscript, and contributed to the intellectual aspect. All authors read and approved the final manuscript.

## Funding

This work was supported by the Ministry of Education of the Republic of Korea and the National Research Foundation of Korea (NRF-2017S1A5B5A02024117) and Institute for Information & communications Technology Planning & Evaluation (IITP) through the Korea government (MSIT) under Grant No. 2021-0-01341 (Artificial Intelligence Graduate School Program, Chung-Ang University).

## Conflict of interest

The authors declare that the research was conducted in the absence of any commercial or financial relationships that could be construed as a potential conflict of interest.

## Publisher’s note

All claims expressed in this article are solely those of the authors and do not necessarily represent those of their affiliated organizations, or those of the publisher, the editors and the reviewers. Any product that may be evaluated in this article, or claim that may be made by its manufacturer, is not guaranteed or endorsed by the publisher.

## References

[ref1] Abuhay T. M. Nigatie Y. G. Kovalchuk S. V. (2018). Towards predicting trend of scientific research topics using topic modeling. Procedia Comput. Sci. 136, 304–310. doi: 10.1016/j.procs.2018.08.284

[ref2] Älgå A. Eriksson O. Nordberg M. (2020). Analysis of scientific publications during the early phase of the COVID-19 pandemic: topic modeling study. J. Med. Internet Res. 22:e21559. doi: 10.2196/21559, PMID: 33031049 PMC7674137

[ref3] Azorín C. (2020). Beyond COVID-19 supernova. Is another education coming? J. Prof. Cap. Community, doi: 10.1108/JPCC-05-2020-0019 [Epub ahead of print].

[ref4] Bas D. Martin M. Pollack C. Venne R. (2020). The impact of COVID-19 on sport, physical activity and well-being and its effects on social development.

[ref5] Batez M. (2021). ICT skills of university students from the faculty of sport and physical education during the COVID-19 pandemic. Sustainability 13:1711. doi: 10.3390/su13041711

[ref6] Borgatti S. P. (2009). “2-mode concepts in social network analysis,” in Encyclopedia of Complexity and Systems Science. *Vol 6*, 8279–8291.

[ref7] Buldú J. M. Antequera D. R. Aguirre J. (2020). The resumption of sports competitions after COVID-19 lockdown: the case of the Spanish football league. Chaos, Solitons Fractals 138:109964. doi: 10.1016/j.chaos.2020.109964, PMID: 32518475 PMC7269962

[ref8] Chan J. Lee A. Lease C. Spurrier N. (2021). Recommencement of sport leagues with spectators at the Adelaide oval during the COVID-19 pandemic: planning, experience, and impact of a globally unprecedented approach. Front. Public Health 9:676843. doi: 10.3389/fpubh.2021.676843 34368052 PMC8345120

[ref9] Chang V. (2018). A proposed social network analysis platform for big data analytics. Technol. Forecast. Soc. Chang. 130, 57–68. doi: 10.1016/j.techfore.2017.11.002

[ref10] Cho S. Shin N. Kwak D. H. Kim A. C. H. Jang W. S. Lee J. S.. (2021). The impact of COVID-19 crisis on major spectator sport industry in the US and South Korea: challenges and outlook. J. Glob. Sport Manag. 2, 1–25. doi: 10.1080/24704067.2021.1936591

[ref11] Contractor F. J. (2022). The world economy will need even more globalization in the post-pandemic 2021 decade. J. Int. Bus. Stud. 53, 156–171. doi: 10.1057/s41267-020-00394-y, PMID: 33551513 PMC7848245

[ref12] Dastani M. Danesh F. (2021). Iranian COVID-19 publications in LitCovid: text mining and topic modeling. Sci. Program. 2021:3315695. doi: 10.1155/2021/3315695

[ref13] Debnath R. Bardhan R. (2020). India nudges to contain COVID-19 pandemic: a reactive public policy analysis using machine-learning based topic modelling. PLoS One 15:e0238972. doi: 10.1371/journal.pone.0238972, PMID: 32915899 PMC7485898

[ref001] Debnath R. Darby S. Bardhan R. Mohaddes K. Sunikka-Blank M. (2020). Grounded reality meets machine learning: A deep-narrative analysis framework for energy policy research. Energy Res. Soc. Sci. 69:101704. doi: 10.1016/j.erss.2020.101704 33145178 PMC7563684

[ref14] DiFiori J. P. Green G. Meeuwisse W. Putukian M. Solomon G. S. Sills A. (2021). Return to sport for north American professional sport leagues in the context of COVID-19. Br. J. Sports Med. 55, 417–421. doi: 10.1136/bjsports-2020-103227, PMID: 32967854

[ref15] Doherty A. Millar P. Misener K. (2022). Return to community sport: leaning on evidence in turbulent times. Manag. Sport Leis. 27, 7–13. doi: 10.1080/23750472.2020.1794940

[ref16] Drewes M. Daumann F. Follert F. (2021). Exploring the sports economic impact of COVID-19 on professional soccer. Soccer Soc. 22, 125–137. doi: 10.1080/14660970.2020.1802256

[ref17] Ebersberger B. Kuckertz A. (2021). Hop to it! The impact of organization type on innovation response time to the COVID-19 crisis. J. Bus. Res. 124, 126–135. doi: 10.1016/j.jbusres.2020.11.051 PMC9754948 36540106

[ref18] Elkhouly R. Tamaki E. Iwasaki K. (2022). Mitigating crowded transportation terminals nearby mega-sports events. Behav. Inform. Technol. 1–17. doi: 10.1080/0144929X.2022.2048890

[ref19] Füzéki E. Groneberg D. A. Banzer W. (2020). Physical activity during COVID-19 induced lockdown: recommendations. J. Occup. Med. Toxicol. 15:25. doi: 10.1186/s12995-020-00278-9 32817753 PMC7422663

[ref20] Giessing L. Kannen J. Strahler J. Frenkel M. O. (2021). Direct and stress-buffering effects of COVID-19-related changes in exercise activity on the well-being of German sport students. Int. J. Environ. Res. Public Health 18:7117. doi: 10.3390/ijerph18137117, PMID: 34281054 PMC8297212

[ref21] González L.-M. Devís-Devís J. Pellicer-Chenoll M. Pans M. Pardo-Ibañez A. García-Massó X.. (2021). The impact of COVID-19 on sport in twitter: a quantitative and qualitative content analysis. Int. J. Environ. Res. Public Health 18:4554. doi: 10.3390/ijerph18094554, PMID: 33923042 PMC8123335

[ref22] Hurley O. A. (2021). Sport cyberpsychology in action during the COVID-19 pandemic (opportunities, challenges, and future possibilities): a narrative review. Front. Psychol. 12:621283. doi: 10.3389/fpsyg.2021.621283, PMID: 33746838 PMC7977283

[ref23] Jiang H. Qiang M. Lin P. (2016). A topic modeling based bibliometric exploration of hydropower research. Renew. Sust. Energ. Rev. 57, 226–237. doi: 10.1016/j.rser.2015.12.194

[ref24] Kaplanidou K. Apostolopoulou A. Cho I. (2021). Sport consumption intentions during a crisis: the COVID-19 pandemic. J. Glob. Sport Manag. 1–23. doi: 10.1080/24704067.2021.1991831

[ref25] Kemp S. Cowie C. M. Gillett M. Higgins R. Hill J. Iqbal Z.. (2021). Sports medicine leaders working with government and public health to plan a ‘return-to-sport’during the COVID-19 pandemic: the UK’s collaborative five-stage model for elite sport. Br. J. Sports Med. 55, 4–5. doi: 10.1136/bjsports-2020-102834 32661129

[ref26] Kim S.-W. Gil J.-M. (2019). Research paper classification systems based on TF-IDF and LDA schemes. HCIS 9, 1–21.

[ref27] Kim D. Seo D. Cho S. Kang P. (2019). Multi-co-training for document classification using various document representations: TF–IDF, LDA, and Doc2Vec. Inf. Sci. 477, 15–29. doi: 10.1016/j.ins.2018.10.006

[ref28] Kumari R. Jeong J. Y. Lee B.-H. Choi K.-N. Choi K. (2021). Topic modelling and social network analysis of publications and patents in humanoid robot technology. J. Inf. Sci. 47, 658–676. doi: 10.1177/0165551519887878

[ref29] Kwon Y. Kwak D. H. (2022). No games to watch: empirical analysis of sport fans’ stress and coping strategies during COVID-19 lockdown. Int. J. Sports Mark. Spons. 23, 190–208. doi: 10.1108/ijsms-02-2021-0053

[ref30] Liu J. Nie H. Li S. Chen X. Cao H. Ren J.. (2021). Tracing the pace of COVID-19 research: topic modeling and evolution. Big Data Res. 25:100236. doi: 10.1016/j.bdr.2021.100236

[ref31] Makarowski R. Piotrowski A. Predoiu R. Görner K. Predoiu A. Mitrache G.. (2020). Stress and coping during the COVID-19 pandemic among martial arts athletes–a cross-cultural study. Arch. Budo 16, 161–171.

[ref32] Maulana N. (2020). Research trends in marketing science before COVID-19 outbreak: a literature review. Manag. Mark. 15, 514–533. doi: 10.2478/mmcks-2020-0030

[ref33] Mimno D. Wallach H. Talley E. Leenders M. McCallum A. (2011). “Optimizing semantic coherence in topic models.” *Paper presented at the Proceedings of the 2011 conference on empirical methods in natural language processing*.

[ref34] Moore M. Reynolds J. Trainor K. Kiefor J. (2022). Pandemics and athletics: how COVID-19 affected sport injury rehabilitation: pandemics and athletics. Sport Soc. Work J. 2, 7–20.

[ref35] Moritz S. Gottschick C. Horn J. Popp M. Langer S. Klee B.. (2021). The risk of indoor sports and culture events for the transmission of COVID-19. Nat. Commun. 12, 1–9. doi: 10.1101/2020.10.28.20221580 34413294 PMC8376924

[ref36] Naraine M. L. Bakhsh J. T. (2022). Optimizing social media engagement in professional sport: a 3-year examination of Facebook, Instagram, and twitter posts. Int. J. Sport Commun. 15, 103–116. doi: 10.1123/ijsc.2021-0079

[ref37] O’callaghan D. Greene D. Carthy J. Cunningham P. (2015). An analysis of the coherence of descriptors in topic modeling. Expert Syst. Appl. 42, 5645–5657. doi: 10.1016/j.eswa.2015.02.055, PMID: 21437291

[ref38] Parker K. Uddin R. Ridgers N. D. Brown H. Veitch J. Salmon J.. (2021). The use of digital platforms for adults’ and adolescents’ physical activity during the COVID-19 pandemic (our life at home): survey study. J. Med. Internet Res. 23:e23389. doi: 10.2196/23389, PMID: 33481759 PMC7857525

[ref39] Pedersen P. M. (2022). Research handbook on sport and COVID-19. Bloomington, IN: Edward Elgar Publishing.

[ref40] Purwanto P. Lumintuarso R. Burhaein E. (2021). Impact of running techniques through the sprint ability in athletes during the COVID-19 pandemic. Int. J. Hum. Mov. Sports Sci. 9, 717–724. doi: 10.13189/saj.2021.090416

[ref41] Qi M. Li P. Moyle W. Weeks B. Jones C. (2020). Physical activity, health-related quality of life, and stress among the Chinese adult population during the COVID-19 pandemic. Int. J. Environ. Res. Public Health 17:6494. doi: 10.3390/ijerph17186494, PMID: 32906604 PMC7558071

[ref42] Ramage D. Rosen E. Chuang J. Manning C. D. McFarland D. A. (2009). “Topic modeling for the social sciences.” in *Paper presented at the NIPS 2009 Workshop on Applications for Topic Models: Text and beyond*.

[ref43] Rashed M. Piorkowski J. McCulloh I. (2019). “Evaluation of extremist cohesion in a darknet forum using ERGM and LDA.” in *Paper presented at the Proceedings of the 2019 IEEE/ACM International Conference on Advances in Social Networks Analysis and Mining*.

[ref44] Ratten V. Thompson A.-J. (2021). “Digital transformation from COVID-19 in small business and sport entities,” in COVID-19 and entrepreneurship. eds. Paul M. P. (Routledge), 54–70.

[ref45] Reisenbichler M. Reutterer T. (2019). Topic modeling in marketing: recent advances and research opportunities. J. Bus. Econ. 89, 327–356. doi: 10.1007/s11573-018-0915-7

[ref46] Romero-Blanco C. Rodríguez-Almagro J. Onieva-Zafra M. D. Parra-Fernández M. L. Prado-Laguna M. D. C. Hernández-Martínez A. (2020). Physical activity and sedentary lifestyle in university students: changes during confinement due to the COVID-19 pandemic. Int. J. Environ. Res. Public Health 17:6567. doi: 10.3390/ijerph17186567, PMID: 32916972 PMC7558021

[ref47] Rueda G. Gerdsri P. Kocaoglu D. F. (2007). “Bibliometrics and social network analysis of the nanotechnology field.” in *Paper presented at the PICMET’07–2007 Portland International Conference on Management of Engineering & Technology*.

[ref48] Ryall E. Edgar A. (2022). “Watching sport during COVID-19” in Philosophy, sport, and the pandemic. eds. Jeffrey P. F. Andrew E. (Routledge), 139–151.

[ref49] Sa H. J. Lee W. S. Lee B. G. (2021). Corona blue and leisure activities: focusing on Korean case. J. Internet Comput. Serv. 22, 109–121. doi: 10.7472/jksii.2021.22.2.109

[ref50] Samuel R. D. Tenenbaum G. Galily Y. (2020). The 2020 coronavirus pandemic as a change-event in sport performers’ careers: conceptual and applied practice considerations. Front. Psychol. 11:567966. doi: 10.3389/fpsyg.2020.567966, PMID: 33071895 PMC7540073

[ref51] Seshadri D. R. Thom M. L. Harlow E. R. Drummond C. K. Voos J. E. (2021). Case report: return to sport following the COVID-19 lockdown and its impact on injury rates in the German soccer league. Front. Sports Act. Living. 3:604226. doi: 10.3389/fspor.2021.604226, PMID: 33681759 PMC7931153

[ref52] Skinner J. Smith A. C. (2021). Introduction: sport and COVID-19: impacts and challenges for the future (Volume 1). Eur. Sport Manag. Q. 21, 323–332. doi: 10.1080/16184742.2021.1925725

[ref53] Smith A. C. Skinner J. (2022). Sport management and COVID-19: trends and legacies. Eur. Sport Manag. Q. 22, 1–10. doi: 10.1080/16184742.2021.1993952

[ref54] Song C.-W. Jung H. Chung K. (2019). Development of a medical big-data mining process using topic modeling. Clust. Comput. 22, 1949–1958. doi: 10.1007/s10586-017-0942-0

[ref55] Sonmez E. Codal K. S. (2021). Determination of research trends in COVID-19 literature using topic model approach. Americas 10, 668–673.

[ref56] Taghizadeh-Hesary F. Akbari H. (2020). The powerful immune system against powerful COVID-19: a hypothesis. Med. Hypotheses 140:109762. doi: 10.1016/j.mehy.2020.109762, PMID: 32388390 PMC7175888

[ref57] Taneja S. L. Passi M. Bhattacharya S. Schueler S. A. Gurram S. Koh C. (2021). Social media and research publication activity during early stages of the COVID-19 pandemic: longitudinal trend analysis. J. Med. Internet Res. 23:e26956. doi: 10.2196/26956, PMID: 33974550 PMC8212965

[ref58] Teare G. Taks M. (2021). Exploring the impact of the COVID-19 pandemic on youth sport and physical activity participation trends. Sustainability 13:1744. doi: 10.3390/su13041744

[ref59] Uslu T. (2022). “Digitalization of recreation and sports in the COVID-19 pandemic period and social identity of exergamers and ePlayers: electronic sports as autochthonous worlds in metaverse,” in Sport management, innovation and the COVID-19 crisis (Routledge), 100–132.

[ref60] Washif J. A. Mohd Kassim S. F. A. Lew P. C. F. Chong C. S. M. James C. (2021). Athlete’s perceptions of a “quarantine” training camp during the COVID-19 lockdown. Front. Sports Act. Living. 2:622858. doi: 10.3389/fspor.2020.622858, PMID: 33521634 PMC7841328

[ref61] Williams J. (2020). Humanity, technology, and nature. Icon 25, 8–28.

[ref62] Zhao W. Chen J. J. Perkins R. Liu Z. Ge W. Ding Y.. (2015). A heuristic approach to determine an appropriate number of topics in topic modeling. BMC Bioinform. 13, 1–10. doi: 10.1186/1471-2105-16-s13-s8 PMC4597325 26424364

[ref63] Zhao W. X. Jiang J. Weng J. He J. Lim E.-P. Yan H.. (2011). “Comparing twitter and traditional media using topic models.” in *Paper presented at the European Conference on Information Retrieval*.

[ref64] Zhu H. Lei L. (2022). A dependency-based machine learning approach to the identification of research topics: a case in COVID-19 studies. Libr. Hi Tech 40, 495–495.

